# The spread of cholera in western Democratic Republic of the Congo is not unidirectional from East–West: a spatiotemporal analysis, 1973–2018

**DOI:** 10.1186/s12879-021-06986-9

**Published:** 2021-12-19

**Authors:** Harry César Ntumba Kayembe, Catherine Linard, Didier Bompangue, Jérémie Muwonga, Michel Moutschen, Hippolyte Situakibanza, Pierre Ozer

**Affiliations:** 1 Service d’Ecologie et Contrôle des Maladies Infectieuses, Département des Sciences de Base, Faculté de Médecine, Université de Kinshasa, Kinshasa, Democratic Republic of the Congo; 2 Département de Sciences Et Gestion de L’environnement, Faculté Des Sciences, Université de Liège, Arlon, Belgium; 3 Département de Géographie, Université de Namur, Namur, Belgium; 4 Chrono-Environnement, UMR CNRS 6249, Université de Franche-Comté, Besançon, France; 5 Département de Biologie Clinique, Faculté de Médecine, Université de Kinshasa, Kinshasa, Democratic Republic of the Congo; 6 Département des Sciences Cliniques, Immunopathologie–Maladies infectieuses et Médecine interne générale, Université de Liège, Liege, Belgium; 7 Département de Médecine Interne, Faculté de Médecine, Université de Kinshasa, Kinshasa, Democratic Republic of the Congo; 8 Département de Parasitologie Et Médecine Tropicale, Faculté de Médecine, Université de Kinshasa, Kinshasa, Democratic Republic of the Congo; 9 Service d’Ecologie et Contrôle des Maladies Infectieuses, Département des Sciences de Base, Faculté de Médecine, Université de Kinshasa, Kin XI, B.P. : 834, Kinshasa, Democratic Republic of the Congo

**Keywords:** Cholera, *Vibrio cholerae*, Epidemic spread, Spatiotemporal analysis, Clusters, Democratic Republic of the Congo

## Abstract

**Background:**

Cholera outbreaks in western Democratic Republic of the Congo (DRC) are thought to be primarily the result of westward spread of cases from the Great Lakes Region. However, other patterns of spatial spread in this part of the country should not be excluded. The aim of this study was to explore alternative routes of spatial spread in western DRC.

**Methods:**

A literature review was conducted to reconstruct major outbreak expansions of cholera in western DRC since its introduction in 1973. We also collected data on cholera cases reported at the health zone (HZ) scale by the national surveillance system during 2000–2018. Based on data from routine disease surveillance, we identified two subperiods (week 45, 2012–week 42, 2013 and week 40, 2017–week 52, 2018) for which the retrospective space–time permutation scan statistic was implemented to detect spatiotemporal clusters of cholera cases and then to infer the spread patterns in western DRC other than that described in the literature.

**Results:**

Beyond westward and cross-border spread in the West Congo Basin from the Great Lakes Region, other dynamics of cholera epidemic propagation were observed from neighboring countries, such as Angola, to non-endemic provinces of southwestern DRC. Space–time clustering analyses sequentially detected clusters of cholera cases from southwestern DRC to the northern provinces, demonstrating a downstream-to-upstream spread along the Congo River.

**Conclusions:**

The spread of cholera in western DRC is not one-sided. There are other patterns of spatial spread, including a propagation from downstream to upstream areas along the Congo River, to be considered as preferential trajectories of cholera in western DRC.

**Supplementary Information:**

The online version contains supplementary material available at 10.1186/s12879-021-06986-9.

## Background

The seventh cholera pandemic (7P), which began in 1961 in the Sulawesi Archipelago (Indonesia), was introduced in Africa in the early 1970s. Since then, it was determined that cholera has been introduced several times into sub-Saharan Africa, causing large epidemics [1]. However, cholera affects the continent heterogeneously. Outbreaks are reported irregularly in most countries, while the disease occurs endemically with a strong annual seasonality in a handful of countries, including the Democratic Republic of the Congo (DRC) [2]. The latter is also one of the world’s major areas of emergence and re-emergence of infectious diseases. Over the last decades, several important outbreaks were reported across the country, including Ebola Virus Disease [3], measles [4, 5], yellow fever [6], and Human African trypanosomiasis [7, 8].

Concerning cholera, the first traces of the disease in the DRC during the 7P were notified in 1973. The southwestern part of the country was affected through cases imported from neighboring Angola [9]. Five years later, cholera was imported from Tanzania to eastern DRC [9, 10], and then spread along the Great Lakes Region (GLR). The largest cholera epidemic occurred in this region, particularly in and around the city of Goma, in 1994 following the Rwandan Genocide and resulted in the deaths of over 50,000 refugees [11]. Between then and 2018, while considering that the 50,000 cholera cases threshold was also exceeded in 2017, the DRC reported over 571,800 cases and 20,900 deaths, accounting for 17% and 21% of African cholera-related morbidity and mortality, respectively [12]. Nevertheless, it should be noted that the proportion of households with access to improved drinking water sources increased from 46% in 2007 to 49% in 2013 and 59% in 2018. In rural areas, this proportion remained limited to less than 35% in 2018, while it reached over 90% in urban areas. Access to improved sanitation facilities also remained low: only 33% of household (54% in urban areas and 16% in rural areas) [13, 14].

Previous epidemiological and ecological studies have identified the lake areas in the GLR of eastern DRC as sources of cholera outbreaks and persistence of *Vibrio cholerae* [2, 15, 16]. From these hotspots, the disease propagates to surrounding areas not yet affected. Moreover, cholera intermittently spreads outside the GLR to main cities in the upstream eastern provinces, and then to downstream western provinces along the Congo River [17–19]. There are also reports of epidemics in some provinces along this axis after Rwandan refugees fled camps in eastern DRC during the second half of the 1990s [20–22]. This could indicate that the pattern of cholera spread in western DRC is mainly one-sided. However, the existence of other modes of propagation in this part of the country should not be excluded. It is therefore more than necessary to highlight the different dynamics of the spread of outbreaks in western DRC. This will allow the establishment of a permanent framework for collaboration between actors at the strategic and political levels aiming at anticipating and controlling them.

Here, the objectives were to summarize major outbreak expansions of cholera in western DRC documented since its introduction in 1973, and to explore alternative patterns of spatial spread in this part of the DRC.

## Methods

### Study setting

**Fig. 1:**
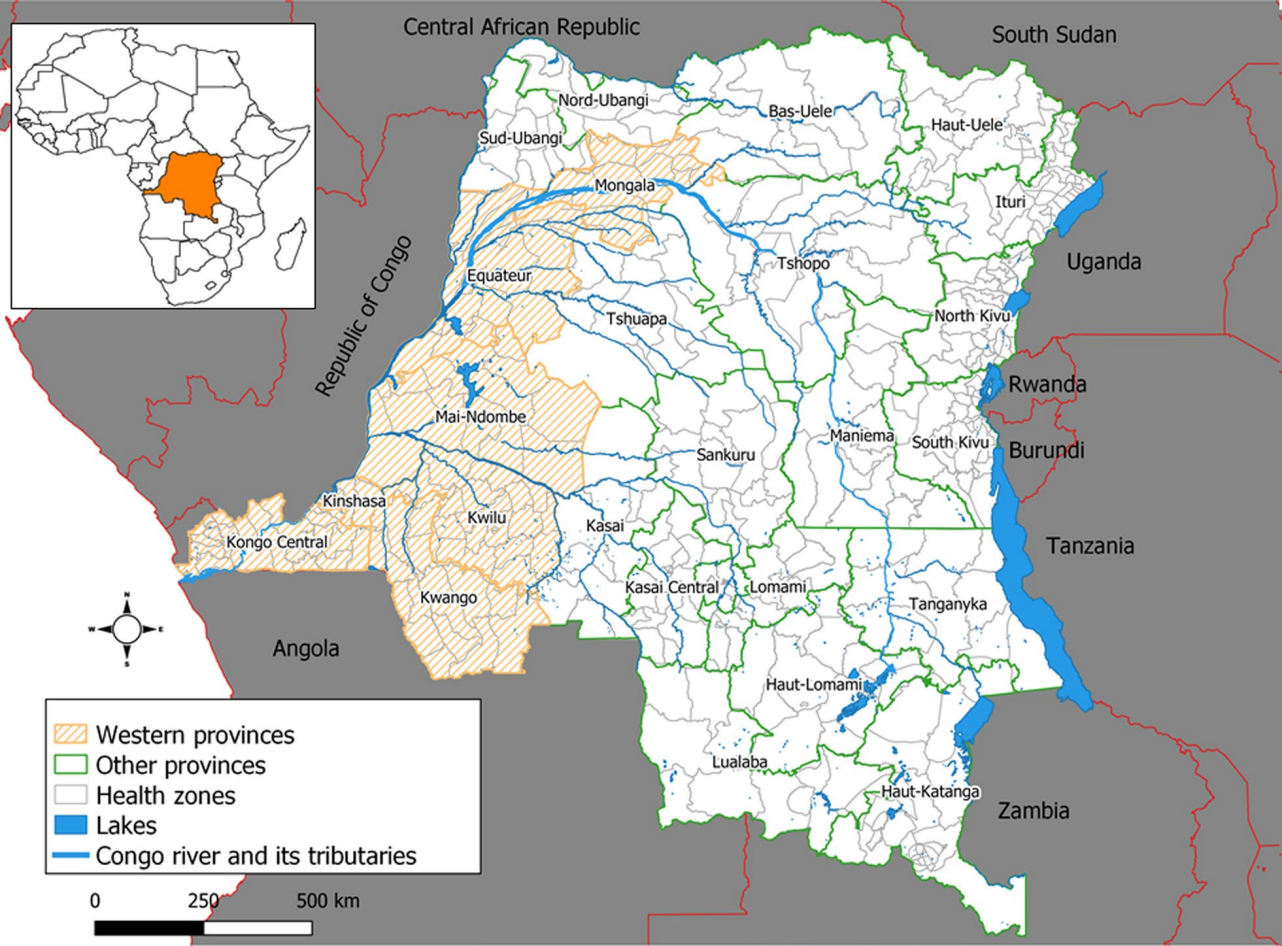
Administrative map of the DRC

This study is focused on the western provinces of DRC which are located in the West Congo Basin. These provinces are among the 26 currently established since the promulgation of the Constitution in 2006 and made effective since 2015. Prior to this period, the DRC had a total of 11 provinces, of which Equateur, Bandundu, Kinshasa, and Bas-Congo (formerly Bas-Zaire) contained the current western provinces considered in this study (Fig. 1) [23].

The western DRC is considered to be affected by cholera outbreaks along the Congo River and then its tributaries. The Congo River generally flows west from Kisangani (Tshopo province), just below the falls, then gradually turns southwest, passing through Mbandaka (Equateur province), joining the Oubangi River, and rushing into the Malebo Pool. The cities of Kinshasa and Brazzaville (Republic of Congo) are located on the opposite banks of the Congo River at the Pool Malebo, where it narrows and forms a number of cataracts created by deep canyons. It then flows in the direction of Kongo Central province, towards Matadi and Boma, then empties into the Atlantic Ocean at the town of Moanda [24].

### Data collection

#### Information on major outbreak expansions of cholera in western DRC documented since its introduction in 1973

We conducted a historical reconstruction of major outbreak expansions of cholera in western DRC since its introduction in 1973 using queries on PubMed, Google scholar, and Google. The following keywords were used: (“cholera” AND “Democratic Republic of the Congo”); (“cholera” AND “Central Africa”). We searched peer-reviewed and non-peer-reviewed articles published in English or French and reports or disease outbreak news from humanitarian agencies focused on the period 1970–2018. Studies and reports or alerts addressing the disease dynamics in western DRC and neighboring countries in the West Congo Basin were included.

#### Cholera data

We obtained data on suspected cholera cases collected at the HZ level through the DRC Integrated Disease Surveillance and Response system (IDSRS) from January 2000 to December 2018. The World Health Organization (WHO) definition of a suspected cholera case is: “In areas where a cholera outbreak has not been declared: Any patient aged 2 years and older presenting with acute watery diarrhea and severe dehydration or dying from acute watery diarrhea. In areas where a cholera outbreak is declared: any person presenting with or dying from acute watery diarrhea” [25]. Each new outbreak is confirmed by culture and isolation of *Vibrio cholerae* O1 from stool samples [26]. In the present study, cholera outbreaks were defined as the periods with the occurrence of at least one case over three consecutive weeks.

#### GIS data

Free open shapefiles of large-scale boundaries of African countries, on the one hand, and the DRC as well as Angola at subnational administrative level on the other hand, were obtained from the open access data platform “The Humanitarian Data Exchange” (https://data.humdata.org/).

#### Data analysis

We cross-referenced information from published articles, epidemiological reports, historical records, and news reports on cholera outbreaks to summarize the major outbreak expansions of the disease in western DRC since its introduction in 1973. Maps of routes of spread were developed using Quantum GIS version 3.8.3.

Based on recent cholera outbreaks that occurred in western DRC, we identified the subperiods to explore other spatial spread patterns than those described in the literature [17, 19]. For each subperiod, we used the Kulldorff’s retrospective space–time permutation scan statistic to detect spatiotemporal clusters of cholera cases using SaTScan software version 9.6 [27]. This model is used to assess which areas are most affected during a relatively short time interval (more or less a year) when the population does not change and to detect the cyclical trend of a disease or to explore unnoticed outbreaks. It requires only the number of cases, with information about the spatial location and time for each case. There is no need for information on the population at risk. Scan statistics are performed using a scanning window, defined as a cylinder with a circular or elliptic base, that moves through space and time, recording the number of observed and expected cases inside the window at each geographical location. Observed cases are compared to expected cases in a cluster if the geographical and temporal locations of all cases are independent of each other. Adjustments are automatically made for both purely spatial and purely temporal clusters. In our case, a cluster was detected in a HZ if, during a specific week, that HZ has a higher proportion of its cases compared to the remaining HZs. The most likely statistically significant cluster, whose window had the highest likelihood, was estimated for each random permutation by 999 Monte Carlo replications of the dataset simulated under the null hypothesis. As Horwood et al. [28], we set the maximum spatial window as a circle with a 125 km radius after identifying very large spatiotemporal clusters containing a number of statistically significant sub-clusters in preliminary analyses.

## Results

We identified a list of 1751 records using the search terms. 208 records were deleted due to duplication and 1523 were excluded according to titles and abstracts or full texts that did not address the dynamics of the spread of cholera in western DRC and neighboring countries in the West Congo Basin. Only 19 studies and reports were considered relevant and detailed in Table 1.

**Table 1:** Summaries of the 21 studies and reports considered as relevant in our study

Citation	Dates of outbreak	Sizes of outbreak	Types of data
Schyns.1979. Cholera in Eastern Zaire, 1978	1973	–	Reports notified to WHO (published in the Weekly Epidemiological Record)
Carme. 1983. L’implantation et l’extension du choléra en Afrique Noire: 1970–1980	1970–1980	–	Reports notified to WHO (published in the Weekly Epidemiological Record)
Rémy & Dejours. 1988. L’Africanisation du choléra	1973	–	Reports notified to WHO (published in the Weekly Epidemiological Record)
Bompangue. 2012. Cholera ante portas–The re-emergence of cholera in Kinshasa after a ten-year hiatus	1996–2001 and 2011	5105 cases and 300 deaths (1996–2001); 6232 cases and 292 deaths (the first 47 weeks of 2011)	Routinely passive surveillance data (1996–2001); Routinely passive surveillance and microbiological data (from all 7 affected DRC provinces including Kinshasa) (2011)
Ingelbeen. 2019. Recurrent cholera outbreaks, Democratic Republic of the Congo, 2008–2017	2008–2017	42,340 suspected cases reported in non endemic provinces (2011–2012 and 2015–2017)	Routinely passive surveillance and microbiological data
Bompangue. 2020. Description of the targeted water supply and hygiene response strategy implemented during the cholera outbreak of 2017–2018 in Kinshasa, DRC	2017–2018	1712 suspected cholera cases, including 53 deaths	Routinely passive surveillance data
Weill. 2017. Genomic history of the seventh pandemic of cholera in Africa	1966–2014	–	Genomic data
Irenge. 2020. Genomic analysis of pathogenic isolates of *Vibrio cholerae* from eastern Democratic Republic of the Congo (2014–2017)	2014–2017	–	Genomic data
Breurec. 2021. Seventh pandemic *Vibrio cholerae* O1 sublineages, Central African Republic	1997–2016	–	Genomic data
Moore. 2018. Epidemiological study of cholera hotspots and epidemiological basins in East and Southern Africa. In-depth report on cholera epidemiology in Angola	2006–2007	67,256 cases and 2,722 deaths	Routinely passive surveillance data
Ministère de la Santé Publique. 2012. Situation épidémiologique du choléra en République Démocratique du Congo en 2011	2006	–	Routinely passive surveillance data
UNICEF. 2011. UNICEF fights "one of the worst ever" cholera outbreaks in West and Central Africa	2011	–	Routinely passive surveillance data
World Health Organization. 2011. Cholera outbreaks in the Democratic Republic of Congo (DRC) and the Republic of Congo	2011	5105 cases and 300 deaths (as of July 20)	Routinely passive surveillance data
UNICEF. 2015. Cholera outbreaks in Central and West Africa: 2015 Regional Update—Week 37	2015	697 cases and 37 deaths (Maniema province)	Routinely passive surveillance data
UNICEF. 2016. Cholera outbreaks in Central and West Africa: 2016 Regional Update—Week 20	2016	Kinshasa (14 cases); Mai Ndombe (32 cases); Equateur (219 cases)	Routinely passive surveillance data
UNICEF. 2016. Cholera outbreaks in Central and West Africa: 2016 Regional Update—Week 46	2016	25,674 cases and 730 deaths	Routinely passive surveillance data
UNICEF. 2017. Cholera outbreaks in Central and West Africa: 2016 Regional Update—Week 52	2016	28,162 cases and 772 deaths (the Congo River basin: DRC, RCA, the Republic of Congo)	Routinely passive surveillance data
UNICEF. 2017. Cholera outbreaks in Central and West Africa: 2017 Regional Update—Week 22	2017	Weeks 18–22: 1371 cases (Mongala province); 1,457 cases (Kongo Central province)	Routinely passive surveillance data
UNICEF. 2017. Cholera outbreaks in Central and West Africa: 2017 Regional Update—Week 52	2017	55,000 cases and 1200 deaths (DRC)	Routinely passive surveillance data

**Fig. 2:**
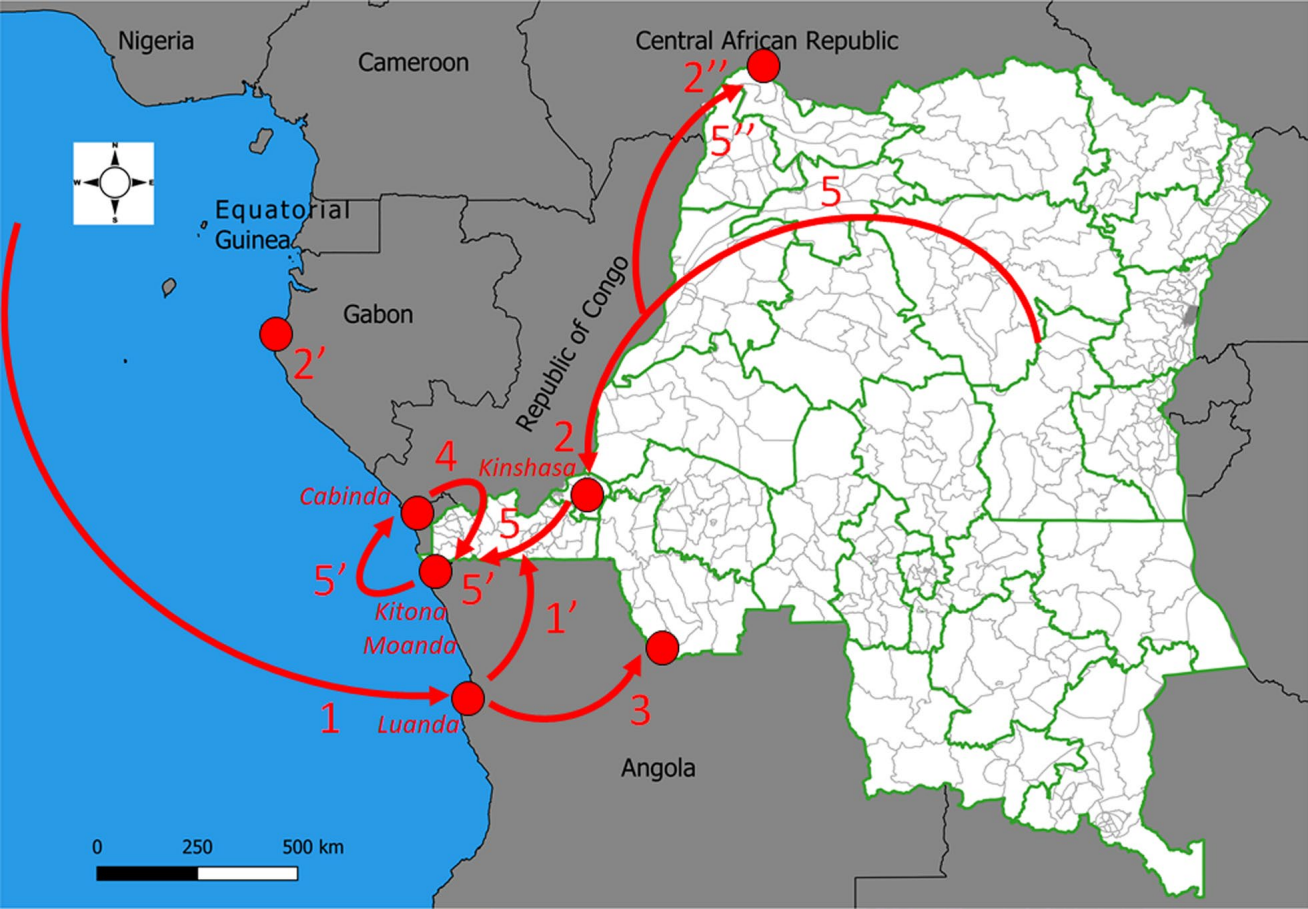
Major outbreak expansions of cholera in western DRC documented since its introduction in 1973

The first epidemics to affect Kongo Central province (formerly Bas-Zaïre) in southwestern DRC were imported from Luanda, the capital of neighboring Angola, in 1973 and 1977 (number 1’, Fig. 2) [9, 29, 30]. These epidemics originated from the wave that hit Angola in December 1971, most likely from the West African coast (number 1, Fig. 2) [1, 29, 30]. Genomic evidence suggests that the strains of *Vibrio cholerae* El Tor that invaded this part of the continent were introduced from South or East Asia to Russia and the Middle East [1].

Figure 2 also summarizes other dynamics of epidemic spread as follows: In 1997, the same *Vibrio cholerae* O1 sublineages were identified in Gabon (number 2’) and southwestern DRC (Kinshasa; number 2) before being identified in Central African Republic (number 2’’) [31]; In 2006, from Luanda (February) to the inland province of Malanje in Angola (April), and then to the former Bandundu province in the DRC in July (number 3) [32, 33]; In 2006, from Cabinda province (Angola) to Kongo Central, particularly in Kitona (19th week) and Moanda (28^th^ week) (number 4) [32, 33]; In 2011–2012 and 2015–2017, from the Great Lakes Region to non-endemic provinces of western DRC (number 5) and then other countries in the West Congo Basin such as Republic of the Congo, Central African Republic (number 5’’) and Angola (particularly in the northern provinces bordering southwestern DRC; number 5’) [17, 19, 32, 34–36]. Westward and cross-border spread in the West Congo Basin has been genetically confirmed [1, 31, 37].

**Fig. 3:**
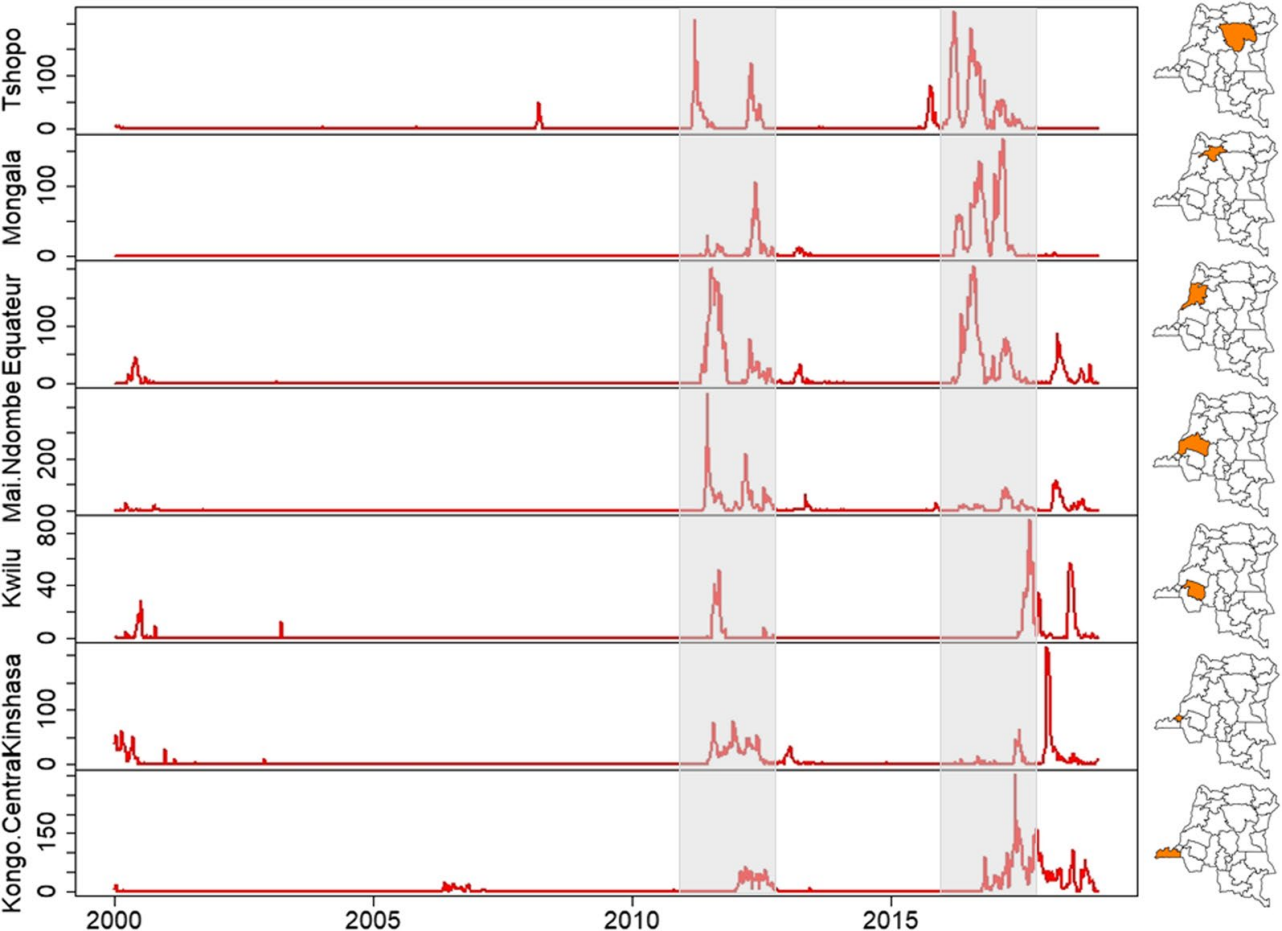
Weekly notification of suspected cholera cases, western provinces of DRC, 2000–2018. From top to bottom: Tshopo, Mongala, Equateur, Maï Ndombe, Kwilu, Kinshasa, Kongo Central. Tshopo province has been included to highlight its non-involvement in the dynamics of cholera outbreaks in western DRC during the periods identified for the spatiotemporal cluster detection analysis. The grey rectangles correspond the periods 2011–2012 and 2016–2017

Figure 3 shows that there were a few small isolated peaks of epidemics in the early 2000s and in 2006, linked to the east–west dynamics initiated in previous years [31] and to cross-border spread from neighboring Angola [32], respectively. Recent cholera outbreaks in provinces of western DRC were reported during 2011–2018. Beyond the westward spread from cholera-endemic areas in the GLR and the recurrence of outbreaks with peaks one year apart described by Ingelbeen et al. in 2011–2012 and 2016–2017 [19], these provinces experienced a very low peak and substantial increases in suspected cholera cases after the first and second periods, respectively. Observed in both November 2012 and 2017 through the following year, it also appears that these outbreaks occurred first in the southwest and then in the upstream provinces.

**Fig. 4:**
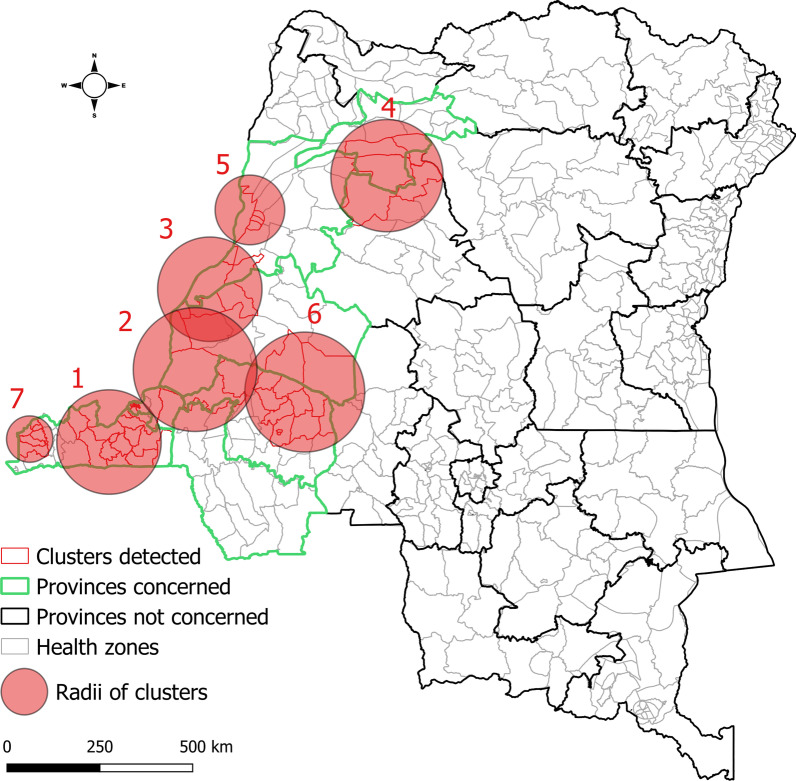
Spatiotemporal clusters of cholera cases, western DRC, week 45, 2012–week 42, 2013

Using SaTScan, the following spatiotemporal clusters were respectively identified during week 45 2012–week 42 2013 (Fig. 4): Kongo Central and western Kinshasa (week 45, 2012–week 4, 2013; cluster 1), eastern Kinshasa and Maï Ndombe (week 7–week 12, 2013; cluster 2), northern Maï Ndombe and southern Equateur (week 7–week 21, 2013; cluster 3), southern Mongala (week 9–week 10, 2013; cluster 4), and central Equateur (week 12–week 23, 2013; cluster 5). Clusters 6 and 7 were detected in southern Maï Ndombe–northern Kwilu (week 22–week 33, 2013) and Kongo Central (week 24–week 42, 2013). See Additional file 1 for details of the clusters.

**Fig. 5:**
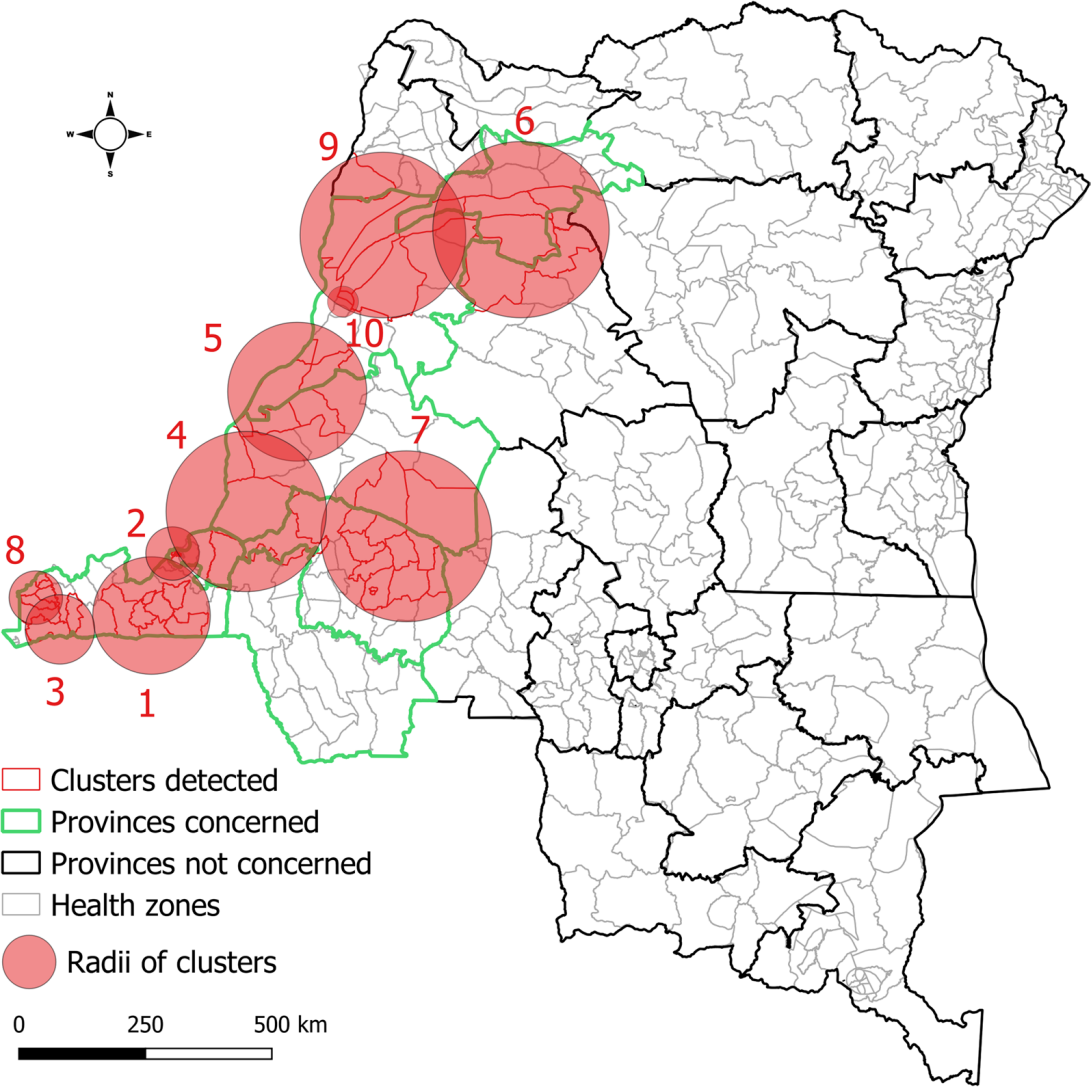
Spatiotemporal clusters of cholera cases, western DRC, week 40, 2017–week 52, 2018

During week 40, 2017–week 52, 2018, spatiotemporal cluster analysis showed the following (Fig. 5): a first cluster detected in Kongo Central (week 40–week 51, 2017; cluster 1), followed by others identified in western Kinshasa (week 51, 2017–week 5, 2018; cluster 2), eastern Kinshasa and Maï Ndombe (week 3–week 15, 2018; cluster 4), northern Maï Ndombe and southern Equateur (week 7–week 21, 2018; cluster 5), Mongala (week 9–week 10, 2018; cluster 6), and northern Equateur (week 42–week 44, 2018; cluster 9). The others were detected in Kongo Central (clusters 3 and 8) and the southern and northern parts of Maï Ndombe and Kwilu, respectively (cluster 7). See Additional file 2 for details of the clusters.

## Discussion

In this study, cross-referencing of published articles, epidemiological reports, historical records, and disease outbreak news of cholera revealed that beyond the spread of cholera from the GLR to western DRC increasingly documented to date [1, 17–19], outbreaks can be imported from neighboring countries into this part of the DRC. The westward spread of cholera is largely considered a significant risk factor of cross-border diffusion in the other countries of the West Congo Basin [1, 31, 37]. However, some cholera outbreaks that occurred in the provinces of southwestern DRC (Kongo Central, Kinshasa, and former Bandundu) were determined to be epidemiologically linked to outbreaks in the bordering northern provinces of Angola [9, 29, 30, 32, 33, 38].

Space–time permutation scan statistical analyses sequentially detected spatiotemporal clusters of cholera cases from southwestern to northwestern provinces in 2012–2013 and 2017–2018. This finding describes a downstream-to-upstream spread of cholera along the Congo River. As major rivers and roads are considered risk factors for cholera transmission [15, 39, 40], substantial population movements across the border and the intensity of interactions at sites of mass gatherings along main roads in southwestern DRC, which is interconnected with upstream provinces via the Congo River, are likely to be associated with such spread dynamics. Note that frequent population movements across the border between the two countries were associated with similar cross-border transmission from Angola to southwestern DRC during the 2015–2016 yellow fever virus outbreak [41].

The results of the present study support the need for a permanent framework to promote cross-border collaboration between the countries within the West Congo Basin. In the field, information exchange and expertise sharing committees are set up in border areas during epidemics. However, these exchanges are infrequent, and even more non-existent in lull periods. The permanent framework for cross-border collaboration is therefore necessary to develop efficient policies and strategies that will allow coordination and implementation of multisectoral prevention and control interventions (previously prioritized); establishment of sustainable funding for cholera surveillance and outbreak emergencies; ensuring access to safe drinking water, sanitation and hygiene, as well as cholera vaccination, if recommended, for vulnerable populations; promoting community awareness of cholera and strengthen the knowledge and skills of local health-care providers [42]. It should be noted that community engagement is the key to successful implementation of interventions. Thus, taking into account local sociocultural sensibilities and practices depends on community-centered health education strategies carried by local stakeholders to build population confidence [43].

There are a number of limitations in this study. First, the use of data on suspected cholera cases. Confirmed cholera data would have smoothed out the probable over or underestimations of the number of cases reported. Also, genomic data from *Vibrio cholerae* O1 isolates would have described more definitive routes of cholera spread [1]. Nevertheless, a recent assessment on the level of adequacy of the weekly reported epidemic-prone diseases monitored by the DRC’s surveillance system demonstrated that data on suspected cholera cases can be used for epidemiological research or public health purposes [44]. Moreover, another assessment of IDSR key performance indicators showed that the DRC figures among the African countries with high coverage of IDSR implementation at subnational level in terms of training, timeliness and completeness of reporting [45]. Second, the lack of cholera data from the national health information system prior to 2000 did not allow to find statistically significant transmission routes for the spread of cholera in western DRC during the corresponding period. However, this does not make the results of this study any less relevant. Our findings have the full merit of providing additional elements of understanding to the current state of knowledge on the spread dynamics of cholera in the DRC. Third, the space–time permutation scan statistic does not utilize information about a background population at risk. Although the overall population of the DRC has doubled over the past two decades [46], population data at the fine geographic scale, such as HZs, correspond to estimates based on a 1984 census with application of a calculated annual growth rate of 1.03% [47]. This poor quality of population data and the relatively short time interval corresponding to the two sub-periods identified to explore alternative patterns of spatial spread of cholera justify the use of the space–time permutation model.

## Conclusions

The present study revealed that the spread of cholera in western DRC is not unidirectional from endemic areas in eastern DRC. Alternative patterns of spatial spread in this part of the country are effectively possible, in particular from downstream to upstream provinces along the Congo River. These patterns of spread should also be considered as preferential trajectories of cholera in the western provinces of the DRC. In addition, this study calls for policies and strategies that focus on cross-border collaboration between the countries in the West Congo Basin to achieve the goals of reducing cholera as a major public health concern by 2030 [48].

## Supplementary Information

**Additional file 1: Table S1.** Detailed spatiotemporal clusters of cholera cases, western DRC, week 45, 2012—week 42, 2013.

**Additional file 2: Table S2.** Detailed spatiotemporal clusters of cholera cases, western DRC, week 40, 2017—week 52, 2018.

## Data Availability

All data generated or analyzed during this study are included in this published article [and its additional information files].

## Abbreviations

7P: The seventh cholera pandemic
DRC: Democratic Republic of the Congo
GLR: Great Lakes Region
HZ: Health zone
IDSRS: Integrated Disease Surveillance and Response system
WHO: World Health Organization
