# Supplementary material for: The spread of cholera in western Democratic Republic of the Congo is not unidirectional from East–West: a spatiotemporal analysis, 1973–2018
Source: BMC Infect Dis. 2021 Dec 19;21:1261. doi: 10.1186/s12879-021-06986-9 (PMC8684622; doi:10.1186/s12879-021-06986-9)
Supplement: Supplementary file 1 — Additional file 1: Table S1. Detailed spatiotemporal clusters of cholera cases, western DRC, week 45, 2012—week 42, 2013. [file 12879_2021_6986_MOESM1_ESM.docx]

**Table S1** Detailed spatiotemporal clusters of cholera cases, western DRC, week 45, 2012 – week 42, 2013.

| **Cluster number** | **Start time** | **End time** | **Radius (km)** | **Observed cases** | **Expected cases** | ***p-value*** |
| --- | --- | --- | --- | --- | --- | --- |
| 1 | Week 45, 2012 | Week 4, 2013 | 109.34 | 1,370 | 511.98 | 10^-17^ |
| 2 | Week 7, 2013 | Week 12, 2013 | 111.29 | 240 | 95.87 | 10^-17^ |
| 3 | Week 7, 2013 | Week 21, 2013 | 97.56 | 1,089 | 499.58 | 10^-17^ |
| 4 | Week 9, 2013 | Week 10, 2013 | 95.03 | 8 | 0.61 | 0.0009 |
| 5 | Week 12, 2013 | Week 23, 2013 | 39.32 | 114 | 29.74 | 10^-17^ |
| 6 | Week 22, 2013 | Week 33, 2013 | 112.26 | 389 | 86.74 | 10^-17^ |
| 7 | Week 24, 2013 | Week 42, 2013 | 46.24 | 699 | 204.68 | 10^-17^ |
