# Supplementary material for: The spread of cholera in western Democratic Republic of the Congo is not unidirectional from East–West: a spatiotemporal analysis, 1973–2018
Source: BMC Infect Dis. 2021 Dec 19;21:1261. doi: 10.1186/s12879-021-06986-9 (PMC8684622; doi:10.1186/s12879-021-06986-9)
Supplement: Supplementary file 2 — Additional file 2: Table S2. Detailed spatiotemporal clusters of cholera cases, western DRC, week 40, 2017—week 52, 2018. [file 12879_2021_6986_MOESM2_ESM.docx]

**Table S2** Detailed spatiotemporal clusters of cholera cases, western DRC, week 40, 2017 – week 52, 2018.

| **Cluster number** | **Start time** | **End time** | **Radius (km)** | **Observed cases** | **Expected cases** | ***p-value*** |
| --- | --- | --- | --- | --- | --- | --- |
| 1 | Week 40, 2017 | Week 51, 2017 | 77.94 | 1,085 | 280.20 | 10^-17^ |
| 2 | Week 51, 2017 | Week 5, 2018 | 11.17 | 850 | 193.14 | 10^-17^ |
| 3 | Week 3, 2018 | Week 15, 2018 | 49.33 | 402 | 172.31 | 10^-17^ |
| 4 | Week 3, 2018 | Week 15, 2018 | 111.29 | 240 | 83.58 | 10^-17^ |
| 5 | Week 7, 2018 | Week 21, 2018 | 97.56 | 1,089 | 430.23 | 10^-17^ |
| 6 | Week 9, 2018 | Week 10, 2018 | 95.03 | 8 | 0.52 | 0.0004 |
| 7 | Week 22, 2018 | Week 33, 2018 | 112.26 | 389 | 86.94 | 10^-17^ |
| 8 | Week 24, 2018 | Week 45, 2018 | 46.24 | 773 | 219.29 | 10^-17^ |
| 9 | Week 42, 2018 | Week 44, 2018 | 107.91 | 17 | 2.64 | 10^-5^ |
| 10 | Week 44, 2018 | Week 44, 2018 | 0 | 28 | 0.75 | 10^-17^ |
